# Digital Mental Health Interventions for Adolescents: An Integrative Review Based on the Behavior Change Approach

**DOI:** 10.3390/children12060770

**Published:** 2025-06-13

**Authors:** Sun Hwa Hong, Tae Kyung Chun, You Jin Nam, Tae Wi Kim, Yong Hyuk Cho, Sang Joon Son, Hyun Woong Roh, Chang Hyung Hong

**Affiliations:** Department of Psychiatry, Ajou University School of Medicine, Suwon 16499, Republic of Korea; sh9@ajou.ac.kr (S.H.H.); eos0306@ajou.ac.kr (T.K.C.); yjnam@ajou.ac.kr (Y.J.N.); rlaxodnl@ajou.ac.kr (T.W.K.); jostradamus@ajou.ac.kr (Y.H.C.); sjsonpsy@ajou.ac.kr (S.J.S.); hansin8607@ajou.ac.kr (H.W.R.)

**Keywords:** adolescent mental health, digital health intervention, behavior change model, prevention, e-health, mobile mental health, digital therapeutics, youth resilience

## Abstract

**Background:** Adolescents are at a critical developmental stage marked by rapid cognitive, emotional, and social changes, making them highly susceptible to mental health issues. Recently, digital health interventions (DHIs) have emerged as innovative and scalable tools for promoting mental well-being in this population. **Methods:** This integrative review was conducted based on comprehensive literature searches of major academic databases, including PubMed, Scopus, Web of Science, and PsycINFO. Studies published between January 2010 and December 2024 were identified using keywords such as “adolescent mental health,” “digital health intervention,” “behavior change model,” “e-health,” “mobile mental health,” and “digital therapeutics.” The inclusion criteria comprised peer-reviewed studies on digital mental health interventions for adolescents that applied, fully or partially, a behavior change approach. Studies targeting adults, interventions without digital technology, the gray literature, and duplicate publications were excluded. **Results:** We examined intervention strategies based on developmental stage prevention, early intervention, and recovery and highlighted key digital components such as accessibility, anonymity, personalization, and continuous monitoring. Furthermore, we analyzed case studies from various countries, including Korea, the United Kingdom, Australia, and Japan, to identify best practices and contextual challenges. **Conclusions:** DHIs rooted in sound psychological theory and ethical design can complement school- and community-based interventions by offering effective personalized support. The practical implications and future directions are discussed.

## 1. Introduction

Adolescence is a critical developmental stage characterized by rapid cognitive, emotional, and social changes during which mental health problems frequently emerge for the first time. According to the World Health Organization [1], the onset of approximately 50% of all mental disorders is before the age of 14 years, and 75% emerge before the age of 24 years. Adolescents exhibit high rates of self-harm, and suicide remains one of the leading causes of adolescent death. Mental health challenges in adolescence are closely associated with a range of adverse outcomes, including poor academic achievement, substance use and abuse, violence, and issues related to sexual and reproductive health [2]. 

If left unaddressed, the mental health problems arising during adolescence may become chronic and exert lasting negative effects throughout adulthood and old age. Therefore, proactive and preventive interventions during adolescence are essential, and a shift toward prevention-oriented strategies is urgently needed [3,4]. However, adolescent mental health care has traditionally relied heavily on treatment-focused and reactive approaches, in which psychiatric diagnoses, pharmacotherapy, and counseling are typically provided only after symptoms become clinically evident [4]. In addition to a lack of early interventions, stigma and limited accessibility contribute to the low rate at which adolescents receive mental health support. Consequently, there is a growing call for a paradigm shift toward delaying the onset of mental illness, preventing its occurrence, and strengthening adolescents’ psychological resources [5]. This shift necessitates moving beyond a treatment-centered model toward an integrated approach that emphasizes mental health promotion and enhancement of psychological resilience [6]. 

To address adolescent mental health from a preventive perspective, interventions should be grounded in a developmental framework that reflects adolescents’ emotional, cognitive, motivational, and behavioral characteristics, referred to as the emotional–cognitive–motivational–behavioral change model. This approach extends beyond symptom relief by fostering self-awareness of emotions (emotional), interpretation of situations (cognitive), reinforcement of internal motivation toward positive change (motivational), and development of the capacity to act on such change (behavioral) [7]. These four psychological domains are highly interconnected during adolescence and play critical roles in mental health development and change. Therefore, an integrated framework that addresses these issues in a unified manner is essential.

Recent advances in digital technology have marked a pivotal shift in the delivery of mental health interventions to adolescents. Adolescents are digital natives and benefit from the anonymity and accessibility offered by mobile applications, online counseling platforms, and AI-based emotional analysis tools [8]. Digital interventions effectively reduce barriers to accessing mental health services and promote voluntary engagement among youth [9]. In particular, the rapid expansion of remote psychological support services following the COVID-19 pandemic has positioned digital interventions at the core of the “new mental health care ecosystem.” Moreover, digital technologies and digital mental health interventions are being increasingly recognized for their scalability and potential to address the rising demand, improve accessibility, and enhance mental health outcomes. The advantages of digital interventions include increased acceptability, enhanced access, efficiency, clinical effectiveness, and the potential for personalized care [10]. Eysenbach [11] was the first to conceptualize the term “e-health,” referring to health services delivered via the Internet and computer-based platforms. Since then, digital mental health interventions have become increasingly prominent [10,11]. An example of a digital health intervention (DHI) is computerized cognitive behavioral therapy (cCBT), which emulates traditional face-to-face CBT sessions through a series of self-paced sequential modules. Recent advancements in programming technologies have enabled the incorporation of gamification and “serious games” into cCBT, enhancing its interactivity and rendering it more suitable for adolescents [12,13,14]. Technological innovations can increase engagement and immersion among adolescents, thereby improving intervention outcomes. Furthermore, the global proliferation of digital devices and rapid expansion in technology use have significantly transformed the landscape of mental health service delivery. Digital platforms now allow for self-monitoring and self-management without temporal or spatial constraints, emerging not just as auxiliary tools but as central media for both assessment and intervention. Given the current communication preferences of adolescents, who often favor social media over face-to-face interactions, digital platforms offer a comfortable and familiar means of expressing psychological distress [15,16]. These changes are increasingly reflected in mental health policies, as digital platforms facilitate self-directed care and real-time monitoring, offering potential advantages in terms of accessibility, efficiency, cost reduction, and effectiveness. User-generated and shared data also open up new possibilities for innovative diagnostic and intervention strategies in clinical settings. When used appropriately, digital mental health platforms that ensure anonymity and accessibility can facilitate rapid responses for adolescents in need of early intervention and prevent chronicity. 

However, to maximize the utility of digital interventions, critical issues must be addressed, including the empirical validation of their effectiveness, protection of personal data, ethical considerations, and applicability across diverse settings. In response to the current adolescent mental health crisis, it is essential to adopt preventive and integrative strategies aligned with contemporary technological and societal shifts. This review explores the structural factors underlying the challenges to adolescent mental health care from the perspective of an integrated behavioral change approach. It also analyzes the theoretical foundations of digital interventions and examines international best practices, thereby offering insights into the applicability and future directions of digital mental health strategies for youth.

To comprehensively explore the existing body of research on digital mental health interventions for adolescents, this study applied an integrative review methodology. Through this approach, diverse research findings were systematically analyzed within a theoretical framework, aiming to derive practical implications for clinical application and future intervention development.

## 2. Main Discussion

### 2.1. Developmental Characteristics of Adolescence

Adolescence is a transitional phase from childhood to adulthood, marked by rapid physical development, cognitive maturation, emotional turbulence, and expanding social relationships. It is generally defined as spanning from around the age of 10 to the early 20s and is characterized by profound psychological instability and multidimensional changes [17]. Four key domains of development are particularly relevant to adolescents’ mental health. 

The first is neurobiological development. During adolescence, the limbic system, which is responsible for emotion and reward processing, undergoes accelerated maturation, whereas, the prefrontal cortex, which governs impulse control, planning, and decision making, matures gradually [18]. According to Casey et al. [19], this imbalance in brain development contributes to impulsive behavior, increased susceptibility to risky actions, and heightened vulnerability to mental health problems.

The second is cognitive development. Adolescents acquire the capacity for abstract and hypothetical thinking along with a growing understanding of themselves and others [20]. However, cognitive flexibility remains incomplete, often resulting in egocentric thought patterns such as the “imaginary audience” and “personal fable” [21]. These tendencies may cause adolescents to become overly sensitive to peer evaluation and social scrutiny, contributing to their emotional instability.

The third is emotional development. Identity formation emerges as a central task in adolescence [22]. The exploration of self-identity can give rise to episodes of depression, anxiety, and confusion as adolescents encounter psychosocial conflict. Emotional distress may be intensified by challenges in peer relationships, the process of separation from parental figures, and navigation of gender and sexual identity [23].

Fourth, social relationships are reconfigured. Adolescents gradually shift from family-centered to peer-centered and socially oriented networks. Peer groups play a critical role in shaping identity, and experiences of peer acceptance or rejection significantly affect emotional well-being [24]. This developmental stage is also marked by an increased desire for social approval and comparison, which is closely linked to adolescents’ social interactions within digital environments such as social media.

### 2.2. Key Components of Mental Health Interventions

Effective adolescent mental health interventions require systematic integration of several core components. In particular, when utilizing digital environments for intervention delivery, four key elements serve as foundations (Table 1).

Accessibility refers to the provision of mental health services that adolescents can utilize without time or location constraints. Given the structured demands of school and daily life, adolescents often face challenges in maintaining consistent access to traditional face-to-face counseling. Digital resources, such as mobile applications, web-based counseling platforms, and 24/7 chatbot services, significantly enhance accessibility. These tools not only allow for more flexible engagement but also facilitate earlier intervention and help reduce treatment avoidance [25]. Second, the provision of a nonjudgmental and safe environment enables adolescents to freely express their emotions and personal challenges. Given their heightened sensitivity to social stigma surrounding mental illness and peer evaluation, adolescents greatly benefit from platforms that ensure anonymity and emotional safety. Digital spaces such as anonymous online communities and emotion diary applications offer a psychologically secure setting, fostering emotional openness and self-expression [26]. Third, person-centered engagement refers to intervention strategies designed to respect adolescents’ autonomy and capacity for self-determination. This approach is operationalized through personalized content recommendations, self-guided therapy modules, and interactive game-based learning systems. Such features position adolescents as active participants, rather than passive recipients, in the intervention process. This shift in role enhances engagement and adherence, ultimately contributing to improved intervention outcomes [27]. Fourth, continuity and monitoring involve the systematic tracking of adolescents’ emotional states and behavioral changes over time, enabling timely detection and response to emerging risks. Tools such as AI-based emotion analysis, regular self-assessment reminders, and visualized emotional dashboards facilitate this function by enhancing intervention consistency and predictability [28].

Collectively, the elements of accessibility, nonjudgmental environment, person-centered engagement, and continuity and monitoring constitute critical pillars for determining the effectiveness and acceptability of adolescent mental health interventions. The integration of digital technologies allows these components to be implemented in practical, sophisticated, and individualized ways. Such a comprehensive framework not only supports short-term intervention success but also provides a foundational structure for long-term emotional stability and self-reliance in youth.

### 2.3. Types and Strategies of Adolescent Mental Health Interventions by Stage

Adolescent mental health interventions require a continuous and structured framework that spans from pre-onset prevention to early detection and response, and finally to recovery and support for self-reliance. In particular, from the perspective of the integrated behavioral change approach, such interventions should not merely aim to reduce symptoms but empower adolescents to recognize their own psychological state, regulate their emotions and behaviors, and ultimately reclaim a sense of autonomy. 

As shown in Table 2, adolescent mental health interventions can be categorized into three main types according to their timing and purpose. Preventive interventions focus on enhancing emotional resilience, increasing mental health literacy, and improving self-management skills prior to the onset of clinically significant symptoms. Representative programs include stress management education, emotion regulation training, and psychoeducational sessions to promote an understanding of mental health.

In recent years, digital tools such as smartphone applications, gamified cognitive behavioral therapy content, and self-assessment tools have been utilized to deliver these interventions in more engaging and scalable formats [29]. For example, Kooth, a UK-based digital platform, offers adolescents opportunities for emotional self-assessment, web-based expressive writing, and peer support through anonymous community forums. It is an integrated preventive tool that enables early engagement and emotional support before clinical treatment becomes necessary [30]. When mental health problems begin to emerge, timely intervention is crucial to prevent symptom escalation and ensure that appropriate treatment and support are delivered at the right time. Early interventions typically involve strategies such as screening at-risk individuals, monitoring emotional states, and linking users to personalized digital counseling services. Recent advances in mobile-based CBT content and AI-powered emotion analysis have significantly improved the efficiency and scalability of early interventions [31]. A representative example in the Republic of Korea is the Maeum-Kkumteo mobile application, which analyzes emotional data from adolescents to recommend professional counseling, and delivers self-regulation content along with automated notifications. This supports rapid and tailored responses during the early stages of distress [32]. Recovery-oriented interventions are essential for adolescents experiencing mental health difficulties. These interventions move beyond symptom management and support adolescents to restore their functional capacity, reintegrate socially, and rebuild their self-esteem. The core components of this approach include strengthening self-management skills, engaging with peer-support networks, exploring career interests, and enhancing self-expression abilities. Recently, digital therapeutics (DTx), which embodies recovery-oriented philosophies, has gained attention as an innovative tool in adolescent mental health care [33]. 

In the United States, reSET-A is a digital therapeutic platform that supports treatment adherence and recovery in adolescents with substance use disorders. In the Republic of Korea, the MARO service provides emotion diary functions, anonymous counseling, and empathy-based community features. It is utilized as a digital resource not only for adolescents in the recovery phase but also for the general public, promoting emotional stability and self-reliance. These examples underscore the importance of stage-specific and individualized approaches in adolescent mental health interventions, from prevention to early response, recovery, and reintegration. In particular, digital interventions are highly effective in minimizing time and space constraints and are designed based on the principles of self-awareness and self-regulation. Therefore, the effectiveness of these interventions depends on their appropriate application, according to the specific needs and developmental stages of adolescents.

### 2.4. Types of Adolescent Mental Health Interventions by Setting

To effectively enhance adolescent mental health, it is essential to implement intervention strategies suited to diverse environments and modes of access. In particular, the school setting, where adolescents spend most of their time; the community setting, which encompasses adolescents and their families; and the digital setting, which is deeply embedded in the everyday lives of the youth, each present unique strengths and limitations. These settings can be used complementarily to maximize the reach, engagement, and effectiveness of mental health interventions (Table 3).

School-based interventions represent the primary setting for adolescent mental health care as schools are where adolescents spend most of their daily lives and form key social relationships with their peers and adults. School settings provide unique opportunities to identify and address emotional and behavioral issues early by leveraging human resources such as teachers, peer groups, and school counselors [34]. Core intervention strategies in this setting include emotion regulation training, social skills development programs, mental health literacy education, and crisis response protocols [35]. These interventions may be integrated into the formal curriculum or delivered through extracurricular activities, group counseling, and special programs. At the preventive level, school-wide mental health education is commonly implemented, whereas early intervention efforts typically involve regular mental health screening to identify high-risk students and refer them to specialized counseling services [36]. School-based interventions are characterized by high accessibility and direct observability of student behaviors and emotional states. However, they also face limitations, including concerns about privacy, the potential for mental health stigma, and limited mental health expertise among school personnel [34,35,36]. Community-based interventions support adolescent mental health through various public and private institutions outside the school system. Key providers include public health centers, mental health welfare centers, youth counseling and welfare centers, and youth shelters. These settings enable a multidisciplinary approach by addressing the ecological context of adolescents, including their families, peers, and community environments [37]. Common strategies include family counseling, crisis intervention, mental health awareness campaigns, peer-led self-help groups, and the development of community-based care networks.

One major advantage of community-based interventions is their ability to reach vulnerable populations such as out-of-school youths, adolescents from low-income families, and those from multicultural backgrounds. However, this model relies heavily on adolescents’ awareness of and voluntary engagement with available services. Additionally, disparities in local resources may lead to unequal access to and inconsistent quality of care across regions. In recent years, the rapid advancement of digital technology has opened new avenues for mental health interventions among adolescents. Adolescents are now accustomed to digital environments and frequently use smartphones, social media, and mobile applications. Consequently, digital-based interventions offer a highly accessible and acceptable strategy for delivering mental health support, particularly for youth who may not otherwise engage with traditional in-person services [38]. 

Digital interventions can be applied across the continuum of adolescent mental health care, including prevention, early intervention, and recovery. Representative modalities include self-assessment tools, AI-based emotion analysis, chatbot counseling, digital CBT content, mood diary applications, and certified digital therapeutics (DTx). In the Republic of Korea, the Maeum-Kkumteo app analyzes adolescents’ emotional states and provides personalized content, along with connections to professional counseling services. In the United Kingdom, Kooth offers a platform for digital expressive writing therapy and peer support communities to foster emotional expression and self-awareness [30]. The key strengths of digital interventions include autonomy, anonymity, and immediate accessibility, which make them particularly valuable for adolescents who may be reluctant to engage in face-to-face care. Digital tools also align well with the digital habits of today’s youth, enhancing acceptability and engagement. However, challenges remain regarding sustained user engagement, data privacy protection, and the need for robust evidence of long-term clinical effectiveness.

### 2.5. Current Landscape of Digital Adolescent Mental Health Services and Analysis from a Behavioral Change Perspective

In this section, the digital adolescent mental health interventions implemented across various countries are analyzed using the integrated behavior change (IBC) framework. For each intervention, we examine how its operational components correspond to the psychological domains of emotional, cognitive, motivational, and behavioral change, as well as how they align with the system-level determinants of the COM-B model (Capability, Opportunity, and Motivation). This integrated mapping allows for a more comprehensive evaluation of both the internal mechanisms and contextual factors influencing each intervention’s design and effectiveness. Digital mental health interventions are being increasingly developed and implemented worldwide, leveraging their natural integration into the everyday lives of adolescents. These interventions are uniquely positioned to align with the routines, preferences, and digital behaviors of the youth. This section examines how digital mental health services are currently designed and operated, and evaluates their effectiveness and limitations through selected representative cases. By analyzing these interventions from a behavioral change perspective, this section seeks to identify the key mechanisms driving user engagement, emotion regulation, and self-management. In doing so, we propose future directions for the development of more adaptive evidence-based digital mental health models tailored to the needs of adolescents.

Recently, digital services for adolescent mental health have garnered increasing attention as a means of overcoming the limitations of traditional offline counseling systems. These platforms offer enhanced accessibility, improved cost-effectiveness, and greater sustainability [39]. Consequently, countries such as the Republic of Korea, the United Kingdom, the United States, Japan, and Germany have developed a range of digital mental health services targeting adolescents and the general public. However, the core functions and approaches of these services vary significantly by country.

Table 4 compares the major national digital mental health services, highlighting differences in aspects such as cost-free availability, online counseling features, AI chatbot-based emotional support, community engagement, and behavior change-focused interventions. To enhance the analytic clarity, Table 4 has been revised to explicitly map the key intervention components of each digital service to both the psychological domains of the IBC model (Emotional, Cognitive, Motivational, and Behavioral) and the system-level components of the COM-B framework (Capability, Opportunity, and Motivation). This mapping allows for a more precise comparative analysis across interventions. In the Republic of Korea, platforms such as MARO, Lime, Mindling, and Mind Café demonstrate a high level of integration by offering free counseling, community-based emotional support, and self-management tools for a wide range of users, including adolescents and high-risk groups [32,40].

The UK’s Kooth, which is integrated with the National Health Service (NHS), provides free mental health services to adolescents, including self-help tools and emotional support. However, the overemphasis on counselor anonymity has raised concerns about the continuity of care and therapeutic trust [41].

From the perspective of the IBC framework, Kooth’s intervention components can be mapped as follows: its self-assessment and journaling functions primarily enhance emotional self-awareness (Emotional domain), while the provision of psychoeducational content supports cognitive restructuring (Cognitive domain). The peer-support forums foster motivation for behavioral change (Motivational domain), while the platform’s 24/7 accessibility, anonymity, and low-barrier entry reflect the Opportunity component within the COM-B model. In the United States, reSET-A focused on behavioral change through simplified digital interventions and initially showed strong accessibility in the growing digital health market. However, operational challenges such as financial instability and limited reach led to its eventual discontinuation [42]. 

In line with the IBC framework, reSET-A functions as a recovery-oriented digital therapeutic (DTx), primarily targeting substance use behaviors via cognitive–behavioral therapy (CBT) modules that directly address both cognitive and behavioral change domains. Personalized feedback mechanisms support the Motivational domain, while the integration of app-based reminders, self-monitoring tools, and structured therapeutic adherence features enhance both the Capability and Opportunity components within the COM-B model. Unlike more preventive interventions such as Kooth or Maeum-Kkumteo, reSET-A focuses on clinically indicated recovery and relapse prevention in adolescents with active substance use disorders. Japan’s KOKOROBO-J is a pilot program focused on suicide prevention using AI-based chatbots. While it represents a progressive initiative within Japan’s hospital-centered mental health system, the limited infrastructure for digital mental health services and lack of preventive intervention mechanisms have restricted its long-term viability [43].

In particular, AI-chatbot services show strong potential in terms of cost-efficiency and accessibility. However, if not integrated with appropriate escalation protocols, they pose risks, particularly when users express severe mental health symptoms. For example, cases in which chatbots respond inappropriately to suicide-related disclosures underscore the need for these tools to operate within collaborative frameworks involving human professionals and be embedded with ethical safeguards and emergency response mechanisms [44]. While many currently operating digital mental health services incorporate key components of the integrated behavioral change approach, such as emotional stabilization, self-awareness, and behavioral activation, gaps remain. These include insufficient tailoring to specific user groups, limited empirical validation, and absence of robust emergency support systems. These limitations, as revealed through the comparative analysis in Table 4, underscore the need for sophisticated digital mental health intervention designs that account for developmental specificity and crisis response capacity in adolescent populations.

To explicitly illustrate the application of the integrated behavior change (IBC) model to the digital mental health interventions discussed in this review, Table 5 summarizes the mapping of intervention components to the three domains of the IBC framework.

The figure illustrates the hierarchical integration of the integrated behavior change (IBC) framework, which synthesizes psychological domains (emotional, cognitive, motivational, and behavioral), system-level COM-B components (capability, opportunity, motivation), and their corresponding digital intervention components (e.g., self-assessment, CBT apps, AI emotion analysis, chatbots). This structure provides a comprehensive framework for understanding digital interventions targeting adolescent mental health.

## 3. Study Limitations and Future Directions

This study conducted a multidimensional analysis of digital mental health interventions for adolescents using an integrative review methodology; however, certain limitations exist. Non-English articles and the gray literature were not included in the search process, and a quantitative meta-analysis could not be performed.

While this review aimed to provide a comprehensive integrative analysis of digital adolescent mental health interventions based on the integrated behavior change (IBC) framework, it is not a fully systematic review. Although multiple major databases were searched, the search process may not have captured all relevant studies, particularly the unpublished gray literature or non-English publications. The inclusion of illustrative national case studies was based on the available literature and expert familiarity, which may introduce potential selection bias and limit generalizability. Moreover, the narrative synthesis approach, while suitable for exploring complex theoretical frameworks, may inherently carry subjective interpretations in mapping interventions to the IBC and COM-B models. Future systematic reviews and meta-analyses may further validate and extend these findings. In this review, while many digital mental health intervention platforms demonstrate promising user acceptance and practical applicability at an early stage, robust empirical validation through randomized controlled trials (RCTs) and large-scale longitudinal studies remains limited, except for Kooth. In particular, for domestic platforms such as MARO and Maeum-Kkumteo, although their implementation is expanding in real-world settings, well-designed clinical trials and comprehensive effectiveness evaluations are still needed. This limitation should be taken into account when interpreting the findings of the present study.

Future research should incorporate a broader range of data sources and consider combining qualitative synthesis with quantitative integration.

## 4. Conclusions

Contemporary adolescents grow up in a digitally mediated environment, where both risk and protective factors for mental health manifest in ways differing from those of previous generations. In response to this shifting landscape, this review sought to establish a framework for adolescent mental health interventions based on the integrated behavior change (IBC) approach. We examined the core components, phase-specific strategies, and implementation settings of adolescent mental health interventions, with an emphasis on digital modalities and representative service models.

**Figure 1:**
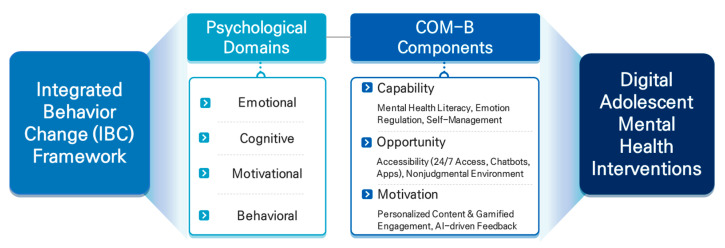
Application of integrated behavior change (IBC) model to digital adolescent mental health interventions.

**Figure 2:**
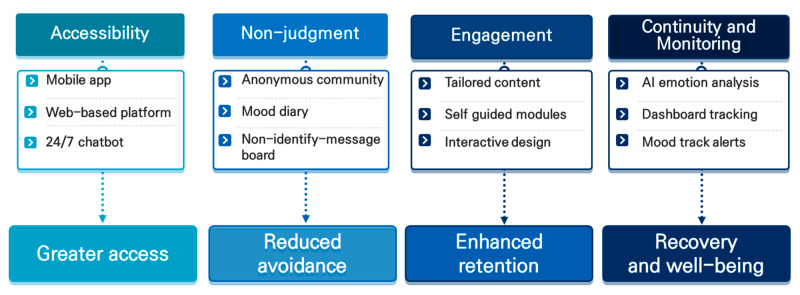
Key components and strategies for digital youth mental health interventions.

Our analysis suggests that digital interventions align well with adolescents’ developmental characteristics and digital lifestyles, demonstrating their potential across the full continuum of care, from prevention to recovery (Figure 1). Various digital tools, including self-assessment platforms, AI-driven emotion recognition, chatbot counseling, mood journaling apps, and digital therapeutics (DTx), serve as effective mechanisms for activating the core elements of the Capability, Opportunity, Motivation–Behavior (COM-B) model. These functions reflect a theoretically coherent and practically adaptable direction for digital intervention design. Case studies from Republic of Korea, the United Kingdom, and Japan further highlight the potential of integrative models that combine digital interventions with school- and community-based approaches. Platforms such as Kooth (UK), KOKOROBO-J (Japan), and Lime (Republic of Korea) illustrate how digital tools extend beyond basic information delivery to provide support for self-understanding, peer support, and professional linkages, thus exemplifying the real-world application of the IBC framework in digital service design. However, several challenges remain to be resolved. Many services lack robust scientific evaluation, and content designed to sustain long-term user engagement and motivation is underdeveloped. To advance digital mental health services as sustainable and effective platforms for adolescent care, there is a need for stronger empirical validation, personalized and interactive content design, and integration into broader support systems that reflect both clinical rigor and user-centered innovation (Figure 2).

Thus, digital interventions for adolescent mental health should not be viewed as standalone solutions but rather as behavior-change mechanisms designed based on sound scientific theory. To serve this function effectively, digital tools must go beyond technological implementation to incorporate developmental understanding, a structured framework of intervention components, and the integration of evidence-based practices. This review demonstrates that this integrated approach is theoretically feasible and practically applicable. These findings may serve as a foundational reference for the development of digitally enabled public mental health strategies targeting youth.

## Figures and Tables

**Table 1:** Core components of mental health interventions and their digital applications.

Component	Description	Example of Digital Application
Accessibility	Providing access to interventions regardless of time and location	Mobile apps, web-based counseling, 24/7 chatbot services
Nonjudgmental environment	Offering a safe space where users can express emotions without fear of stigma or evaluation	Anonymous online communities, emotion diary applications
Person-centered engagement	Designing interventions that emphasize autonomy and self-direction for adolescents	Personalized content recommendations, self-guided therapy modules
Continuity and monitoring	Enabling long-term tracking of emotional states and timely responses to changes	AI-based emotion analysis, automated alerts, risk detection system

**Table 2:** Stage-based approaches to adolescent mental health and representative applications.

Type (Stage)	Objective	Strategic Approach	Applied Technology	Representative Example
Preventive (Pre-onset)	Minimizing risk factors and enhancing protective factors	- Mental health literacy education - Emotion regulation training - Stress management strategies	- Mobile applications - Self-assessment tools - CBT-based digital content	Kooth (UK), MindMatters (Australia)
Early	Prevention of symptom worsening, early detection, and timely intervention	- Risk group screening - Emotion monitoring - Tailored counseling connection	- AI-based emotion analysis - App-based screening tools - Digital CBT	Maeum-Kkumteo (Republic of Korea), FOCUS (Finland)
Recovery & Empowerment-focused	Functional recovery, autonomy enhancement, and social reintegration	- Self-management training - Peer support - Restoration of self-esteem	- DTx platforms - Emotion diary - Online counseling/communities	reSET-A (USA) MARO (Republic of Korea)

**Table 3:** Strengths and limitations of adolescent mental health interventions by setting.

	School	Community	Digital
**Environment**	In-school education and counseling systems	Public health centers, mental health centers, local welfare institutions	Smartphone applications, web-based platforms
**Strategies**	Mental health literacy education, emotion regulation training, screening	Family counseling, crisis intervention, community-based networking	Self-assessment tools, chatbots, AI-based emotion analysis, CBT apps, digital therapeutics (DTx)
**Target**	General student population, high-risk youth within the school system	Vulnerable youth, out-of-school adolescents, families included	All adolescents with access to digital devices
**Strengths**	High accessibility, early identification through educators	Ecological approach, family engagement, multidisciplinary intervention possible	No time or location constraints, anonymity, high autonomy
**Limitations**	Risk of stigma, limited mental health expertise among teachers	Regional disparities in resources, low voluntary engagement	Privacy concerns, difficulty sustaining engagement
**Examples**	School-based mental health education programs, school counseling rooms	Youth counseling centers, regional mental health and welfare programs	Kooth (UK), Maeum-Kkumteo (Republic of Korea), reSET-A (USA)

**Table 4:** Application of the integrated behavior change (IBC) model and COM-B framework to representative digital adolescent mental health services.

Service	Country	Psychological Domains (IBC)	COM-B Components	Evidence Level
Kooth	UK	Emotional (self-awareness via journaling), Cognitive (psychoeducation), Motivational (peer support)	Capability (self-guided modules), Opportunity (24/7 access, anonymity), Motivation (peer engagement)	Pilot evaluation (Stevens et al., 2022 [30])
Maeum-Kkumteo	Korea	Emotional (emotion diary), Cognitive (self-assessment feedback), Motivational (automated coaching)	Capability (self-assessment), Opportunity (mobile platform access), Motivation (personalized reminders)	Early service reports, no published RCT
MARO	Korea	Emotional (emotion tracking), Cognitive (self-expression via emotion diary), Motivational (empathy-based community)	Capability (emotion monitoring), Opportunity (anonymous community), Motivation (personalized coaching)	Internal reports, no published RCT
reSET-A	USA	Cognitive (CBT modules), Behavioral (relapse prevention training), Motivational (adherence feedback)	Capability (skills training), Opportunity (app-based monitoring), Motivation (personalized feedback)	Approved DTx, clinical trials available

**Table 5:** Adolescent digital mental health interventions mapped to IBC components.

IBC Component	Intervention Focus	Digital Examples
**Capability**	Mental health literacy, emotion regulation training, self-management skills	Online psychoeducation, CBT-based mobile apps, emotion diary tools
**Opportunity**	Accessibility, anonymity, nonjudgmental environments	24/7 chatbot services, mobile apps, anonymous peer communities
**Motivation**	Personalized content, self-directed engagement, gamification	AI-based emotion analysis, personalized feedback, gamified CBT modules

## Data Availability

The original contributions presented in this study are included in the article.

## Abbreviations

The following abbreviations are used in this manuscript:

DHI	Digital health intervention
CBT	Cognitive behavior therapy
cCBT	Computerized cognitive behavior therapy
DTx	Digital therapeutics

## References

[1] World Health Organization (2021). Adolescent Mental Health.

[2] Verhulst F.C., Achenbach T.M., Van Der Ende J., Erol N., Lambert M.C., Leung P.W.L., Silva M.A., Zilber N., Zubrick S.R. (2003). Comparisons of problems reported by youths from seven countries. Am. J. Psychiatry.

[3] Kessler R.C., Berglund P., Demler O., Jin R., Merikangas K.R., Walters E.E. (2005). Lifetime prevalence and age-of-onset distributions of DSM-IV disorders in the National Comorbidity Survey Replication. Arch. Gen. Psychiatry.

[4] Patel V., Flisher A.J., Hetrick S., McGorry P. (2007). Mental health of young people: A global public-health challenge. Lancet.

[5] Costello E.J., Foley D.L., Angold A. (2006). 10-year research update review: The epidemiology of child and adolescent psychiatric disorders: II. Developmental epidemiology. J. Am. Acad. Child Adolesc. Psychiatry.

[6] Mrazek P.J., Haggerty R.J. (1994). Reducing Risks for Mental Disorders: Frontiers for Preventive Intervention Research.

[7] Prochaska J.O., DiClemente C.C. (1983). Stages and processes of self-change of smoking: Toward an integrative model of change. J. Consult. Clin. Psychol..

[8] Hollis C., Morriss R., Martin J., Amani S., Cotton R., Denis M., Lewis S. (2015). Technological innovations in mental healthcare: Harnessing the digital revolution. Br. J. Psychiatry.

[9] Johnson K.R., Fuchs E., Horvath K.J., Scal P. (2015). Distressed and looking for help: Internet intervention support for arthritis self-management. J. Adolesc. Health.

[10] Hollis C., Martin J., Amani S., Cotton R., Denis M., Lewis S. (2017). Digital health interventions for children and young people with mental health problems: A systematic and scoping review. Eur. Child Adolesc. Psychiatry.

[11] Eysenbach G. (2001). What is e-health?. J. Med. Internet Res..

[12] Fleming T., Bavin L., Stasiak K., Hermansson-Webb E., Merry S.N., Cheek C., Lucassen M., Lau H.M., Pollmuller B., Hetrick S. (2014). Serious games for the treatment or prevention of depression: A systematic review. Rev. Psicopatol. Psicol. Clin..

[13] Lucassen M.F., Merry S.N., Hatcher S., Frampton C.M. (2015). Rainbow SPARX: A novel approach to addressing depression in sexual minority youth. Cogn. Behav. Pract..

[14] Fleming T., Lucassen M., Stasiak K., Sutcliffe K., Merry S. (2021). Technology matters: SPARX–computerised cognitive behavioural therapy for adolescent depression in a game format. Child Adolesc. Ment. Health.

[15] Berry N., Lobban F., Emsley R., Bucci S. (2016). Acceptability of interventions delivered online and through mobile phones for people who experience severe mental health problems: A systematic review. J. Med. Internet Res..

[16] Batterham P.J., Calear A.L. (2017). Preferences for internet-based mental health interventions in an adult online sample: Findings from an online community survey. JMIR Ment. Health.

[17] Blakemore S.J., Mills K.L. (2014). Is adolescence a sensitive period for sociocultural processing?. Annu. Rev. Psychol..

[18] Steinberg L. (2005). Cognitive and affective development in adolescence. Trends Cogn. Sci..

[19] Casey B.J., Getz S., Galvan A. (2008). The adolescent brain. Dev. Rev..

[20] Piaget J. (1972). Intellectual evolution from adolescence to adulthood. Hum. Dev..

[21] Elkind D. (1967). Egocentrism in adolescence. Child Dev..

[22] Erikson E.H. (1968). Identity: Youth and Crisis.

[23] Laursen B., Collins W.A., Lerner R.M., Steinberg L. (2009). Parent–child relationships during adolescence. Handbook of Adolescent Psychology.

[24] Brown B.B., Larson J., Lerner R.M., Steinberg L. (2009). Peer relationships in adolescence. Handbook of Adolescent Psychology.

[25] Clarke A.M., Kuosmanen T., Barry M.M. (2015). A systematic review of online youth mental health promotion and prevention interventions. J. Youth Adolesc..

[26] Biddle L., Gunnell D., Sharp D., Donovan J.L. (2007). Factors influencing help seeking in mentally distressed young adults: A cross-sectional survey. Br. J. Gen. Pract..

[27] Torous J., Myrick K.J., Rauseo-Ricupero N., Firth J. (2020). Digital mental health and COVID-19: Using technology today to accelerate the curve on access and quality tomorrow. JMIR Ment. Health.

[28] Larsen M.E., Nicholas J., Christensen H. (2019). A systematic assessment of smartphone tools for suicide prevention. PLoS ONE.

[29] Santre S. (2022). Mental health promotion in adolescents. J. Indian Assoc. Child Adolesc. Ment. Health.

[30] Stevens M., Farías J.C., Mindel C., D’Amico F., Evans-Lacko S. (2022). Pilot evaluation to assess the effectiveness of youth peer community support via the Kooth online mental wellbeing website. BMC Public Health.

[31] Kessler R.C., Berglund P., Demler O., Jin R., Merikangas K.R., Walters E.E. (2007). Lifetime prevalence and age-of-onset distributions of mental disorders in the World Health Organization’s World Mental Health Survey Initiative. World Psychiatry.

[32] Lee S., Yoon J., Cho Y., Chun J. (2023). Systematic review of extended reality digital therapy for enhancing mental health among South Korean adolescents and young adults. Korean J. Child Adolesc. Psychiatry.

[33] Slade M. (2009). Personal Recovery and Mental Illness: A Guide for Mental Health Professionals.

[34] Weare K., Nind M. (2011). Mental health promotion and problem prevention in schools: What does the evidence say?. Health Promot. Int..

[35] Larsson K.H., Zetterqvist M. (2024). An emotion regulation skills training for adolescents and parents: Perceptions and acceptability of methodological aspects. BMC Psychiatry.

[36] Mansfield R., Patalay P., Humphrey N. (2020). A systematic literature review of existing conceptualisation and assessment measures of mental health literacy in adolescent research: Current challenges and inconsistencies. Int. J. Environ. Res. BMC Public Health.

[37] Bronfenbrenner U. (1979). The Ecology of Human Development: Experiments by Nature and Design.

[38] Torous J., Wisniewski H., Liu G., Keshavan M. (2018). Mental health mobile phone app usage, concerns, and benefits among psychiatric outpatients: Comparative survey study. JMIR Ment. Health.

[39] Rickwood D., Deane F.P., Wilson C.J., Ciarrochi J. (2007). Young people’s help-seeking for mental health problems. Adv. Ment. Health.

[40] Korea National Center for Mental Health (2022). National Mental Health Survey of Korea–Child & Adolescent 2022.

[41] Kooth Kooth Annual Impact Report. https://connect.kooth.com.

[42] Sawyer-Morris G., Wilde J.A., Molfenter T., Taxma F. (2023). Use of digital health and digital therapeutics to treat SUD in criminal justice settings: A review. Curr. Addict. Rep..

[43] Kanata T., Takeda K., Fujii T., Iwata R., Hiyoshi F., Iijima Y., Nakao T., Murayama K., Watanabe K., Kikuchi T. (2024). Gender differences and mental distress during COVID-19: A cross-sectional study in Japan. BMC Psychiatry.

[44] Fitzpatrick K.K., Darcy A., Vierhile M. (2017). Delivering cognitive behavior therapy to young adults with symptoms of depression and anxiety using a fully automated conversational agent (Woebot): A randomized controlled trial. JMIR Ment. Health.

